# Sex-Specific Incidence Rates and Risk Factors for Hypertension During 13 Years of Follow-up: The Tehran Lipid and Glucose Study

**DOI:** 10.5334/gh.780

**Published:** 2020-04-08

**Authors:** Samaneh Asgari, Seyyed Saeed Moazzeni, Fereidoun Azizi, Hengameh Abdi, Davood Khalili, Monir Sadat Hakemi, Farzad Hadaegh

**Affiliations:** 1 Metabolic Disorders Research Center, Research Institute for Endocrine Sciences, Shahid Beheshti University of Medical Sciences, Tehran, IR; 2 Nephrology Ward of Shariati Hospital, Tehran University of Medical Sciences, Tehran, IR; 3 Endocrine Research Center, Research Institute for Endocrine Sciences, Shahid Beheshti University of Medical Sciences, Tehran, IR; 4 Department of Epidemiology and Biostatistics, Research Institute for Endocrine Sciences, Shahid Beheshti University of Medical Sciences, Tehran, IR

**Keywords:** hypertension, incidence, risk factors

## Abstract

**Background:**

Hypertension, with a prevalence of 25.6% is a serious public health concern in Iran.

**Objective:**

To investigate the population-based incidence of hypertension and its potential risk factors in Tehranian adults during a median follow-up of 13.1 years.

**Methods:**

A total of 6,533 non-hypertensive participants (women = 3,639), aged ≥20 years participated in the study. Crude and age-standardized incidence rates per 1000 person-years were calculated for each sex, separately. Multivariable Cox proportional hazard models were used to estimate hazard ratios (HRs) and 95% confidence intervals (CI) for all potential risk factors.

**Results:**

The crude and age-standardized incidence rates (95% CI) of hypertension per 1000 person-years were 29.7 (27.8–31.6) and 34.9 (32.5–37.4) among men and 25.8 (24.3–27.3) and 38.7 (35.5–42.0) among women, respectively. The incidence rate of hypertension in younger age groups was higher among men. However, after the 4^th^ decade, the incidence rate was higher among women. Significant interactions of sex with age groups, body mass index categories, marital status, hypertriglyceridemia and glycemic categories were found in multivariable analyses (all p-values < 0.05). In the multivariable model, the risk in both sexes was found to be significantly associated with older age, obesity, and normal or high normal blood pressure (BP). Moreover, factors such as being overweight [HR: 1.20 (1.00–1.44)], former smoking [2.15 (1.52–3.04)], hypertriglyceridemia [1.23 (1.06–1.43)] and pre-diabetes status [1.19 (1.02–1.39)] were significant predictors of incident hypertension among women. Central obesity was found to be a significant predictor among men [1.26 (1.03–1.54)]. The optimism-corrected Harrell’s C index (95% CI) in the categorical adjusted model was 0.75 (0.74–0.79) among men and 0.75 (0.74–0.76) among women.

**Conclusion:**

In the Tehranian population, nearly 2.7% of total participants (3% of men and 2.6% of women) develop hypertension each year. Obesity and high BP levels are the main modifiable risk factors in both sexes. Hypertriglyceridemia, prediabetes and former smoking are risk factors for hypertension among women.

## Introduction

High blood pressure (hypertension) is a serious public health concern and is responsible for 9.4 million deaths annually worldwide [1]. National data from an Iranian survey showed that among adults aged 25–70 years, the prevalence of hypertension and pre-hypertension were 25.6% and 39.8%, respectively [2]. Moreover, during six years of follow-up of non-diabetic Iranian men and women, sex-specific incidence rates of hypertension were reported to be 30.9 and 29.3 per 1000 person-years, respectively [3].

Behavioral risk factors such as low physical activity (PA), unhealthy diet and smoking in addition to metabolic risk factors such as obesity, type 2 diabetes mellitus (T2DM) and hyperlipidemia increase the risk of hypertension development [4,5]. Additionally, socioeconomic factors such as urbanization, air pollution, and education contribute to the rising prevalence of hypertension in some populations [4,6]. Only a few studies [4] have investigated the effect of modification by sex on the associations between different risk factors and hypertension incidence. Even though hypertension mostly affects populations in low- and middle-income countries [1,7], data on the incidence of hypertension in these regions is very scarce [3,8,9].

The aim of the current study is not only to extend the duration of our short-term study [3] to the incidence rates of hypertension during a 13-year follow-up among women and men. We also assess the sex specific risk factors associated with hypertension in the oldest cohort of the Middle East and North Africa (MENA) region, namely the Tehran Lipid and Glucose Study (TLGS).

## Materials and methods

### Study design and population

The TLGS is a population-based longitudinal study conducted on individuals aged ≥3 years living in the urban area of Tehran, the capital of Iran. This study aimed to determine the prevalence and incidence of non-communicable diseases and their related risk factors. It also looked at developing a healthy lifestyle to counteract these risk factors. TLGS enrollment was carried out in two phases, the first of which was from January 31, 1999 to July 03, 2001, with a second enrollment phase from October 20, 2001 til September 22, 2005. Data collection is planned to continue for at least 20 years with approximately 3-year intervals (i.e. phase III: 2005–2008, phase IV: 2008–2011, phase V: 2011–2014, and phase VI: 2015–2018). The design and registration of the TLGS have been described previously [10].

Among a total of 12617 participants aged ≥20 years [10219 individuals from phase I and 2398 new participants from phase II], we excluded subjects with prevalent hypertension (n = 1694), those receiving anti-hypertensive medications (n = 959), those with prevalent cardiovascular disease (CVD) (n = 239) and pregnant women (n = 95). Furthermore, from the remaining 9630 individuals, those with missing data regarding body mass index (BMI), waist circumference (WC), triglyceride levels (TG), creatinine (Cr), fasting plasma glucose (FPG), 2-hour post-challenge plasma glucose (2h-PCPG), high-density lipoprotein cholesterol (HDL-C), total cholesterol (TC), systolic blood pressure (SBP), diastolic blood pressure (DBP), smoking status, education level and marital status at baseline (n = 1767) were excluded by considering overlapping features. After excluding subjects without any follow-up measurements after baseline recruitment (n = 1330), a total of 6533 participants (2894 men and 3639 women) were followed until April 2018 (the 6th examination cycle) for the current study analyses.

The ethics committee of the Research Institute for Endocrine Sciences of Shahid Beheshti University of Medical Sciences approved the study proposal, and written informed consent was obtained from all participants.

### Clinical and laboratory measurements

Body measurements of the study participants (weight and height) were recorded with shoes removed and while wearing light clothing. Weight was measured to the nearest 100 grams. Height was measured in a standing position, using a tape measure, while shoulders were in normal alignment. WC was measured with light clothing at the level of the umbilicus [10].

A standard questionnaire was used to collect information on demographic data, personal and familial history of CVD, medical and medication history, smoking habits, education level and marital status.

Based on the TLGS design [10], two measurements of SBP and DBP were taken on the right arm after a 15-min rest in a sitting position. The mean of two measurements was considered as the subject’s blood pressure (BP). A blood sample was taken following a 12–14 hour overnight fasting from all study participants between 7:00 and 9:00 AM. Details for laboratory measurements including FPG, TC, HDL-C, and creatinine were reported elsewhere [10]. All blood analyses were carried out in the TLGS research laboratory on the day of blood collection.

### Definition of terms

General obesity was classified into three groups: <25 kg/m^2^ (normal), ≥25 and <30 kg/m^2^ (overweight) and ≥30 kg/m^2^ (obese). Central obesity was defined as WC ≥95 cm for both sexes [11]. Age was categorized into 5 groups: ‘20s, ‘30s, ‘40s, ‘50s and ≥60 years. Education was sorted into 3 groups: formal education lasting less than 6 years, 6–12 years and greater than 12 years. Marital status was defined as either single, married or widowed/divorced. Prior diagnosed CVD in female first-degree blood relatives aged <65 years or male first-degree blood relatives aged <55 years was considered a positive family history of premature CVD for the participant. Smoking status was categorized into three groups; current smokers (who smoke cigarettes daily or occasionally), former smokers (those who used to smoke) and never smokers. We defined low physical activity as being physically active for fewer than three days per week according to the TLGS. This was measured using the Lipid Research Clinic (LRC) questionnaire for participants who were enrolled in phase I. For those participants who were recruited in phase II, the Modifiable Activity Questionnaire (MAQ) was used and individuals with fewer than 600 MET (metabolic equivalent task)-minutes per week were categorized as being in the low physical activity group [10,12]. Estimated Glomerular Filtration Rate (eGFR; mL/min per 1.73 m^2^) was estimated using the Chronic Kidney Disease Epidemiology Collaboration (CKD-EPI) abbreviated prediction equation [13]. CKD was defined as an eGFR of less than 60 mL/min per 1.73 m^2^ for longer than 3 months. Diabetes was defined as having FPG≥7 mmol/L and/or 2–hPCPG ≥11.1 mmol/L or the use of anti-diabetic medications. Moreover, prediabetes status was defined as the presence of 5.6≤FPG<7 mmol/L or 7.7≤2h-PCPG<11.1 mmol/L. Hypercholesterolemia was defined as having TC≥5.2 mmol/L or use of lipid-lowering medications. Moreover, with respect to the Joint Interim Statement [14], hypertriglyceridemia was defined as TG≥1.695 mmol/L and low HDL-C was defined as HDL-C<1.036 mmol/L for men and <1.295 mmol/L for women, or taking lipid-lowering medications. According to the recommendation of the 2018 ESC/ESH Guidelines for the management of arterial hypertension [15], baseline blood pressure was classified into four groups; optimal: SBP<120 mmHg and DBP<80 mmHg, normal: SBP 120–129 mmHg and DBP 80–84 mmHg, high normal: SBP 130–139 mmHg and/or DBP 85–89 mmHg and hypertension: SBP≥140 mmHg or DBP≥90 mmHg or using anti-hypertensive medications.

### Statistical analysis

Baseline characteristics of the study population were described as mean (standard deviation: SD) values for continuous variables, and as frequencies (%) for categorical variables. For covariates with a skewed distribution (e.g. TG), the median (interquartile range: IQR) was reported. Comparison of the baseline characteristics of respondents (those with complete data at baseline who entered the study) and non-respondents (those with missing data at baseline or lost to follow-up) was done using the Student’s t-test for normal distributed continuous variables, the Chi-squared test for categorical variables and the Mann-Whitney U statistic for skewed and ordered variables.

The crude incidence rate (95% confidence interval; CI) was calculated by dividing the number of new cases of hypertension by person-years at risk for each sex and the whole population. Age-standardized incidence rates (ASRs) were calculated using Segi’s world standard population [16]. Univariable and multivariable Cox proportional hazards models were used to evaluate the associations of potential risk factors with the incidence of hypertension. The event date was defined as the date of incident hypertension. Those who met the following criteria were considered to be censored: leaving the residential area, death, loss to follow-up or end of follow-up. For individuals with incident hypertension, survival time was defined as the mid-time between the entered date and the event date. Additionally, for the censored participants, the survival time was defined as the difference between the entered date and the last available follow-up date.

Risk factors including age, BMI, WC, baseline SBP/DBP, FPG, 2h-PCPG, TC, TG, HDL-C, eGFR, physical activity, family history of premature CVD, education, smoking, marital status, glucose-lowering medication and lipid-lowering medication, with a p-value < 0.25 in univariable analysis were selected to enter the multivariable model. To be more applicable in clinical decision-making [17], all analyses were also performed by categorizing the above-mentioned potential risk factors in our data analysis. Schoenfeld’s global test of residuals was used to test the proportionality assumption of the multivariable Cox regression. To be sure about the event classification ability of the suggested variables, we calculated Harrell’s C index, and in order to consider optimization, we reported optimism-corrected C-index (95% CI) using bootstrap resampling with 1000 replications [18,19]. We also reported the interaction of sex with potential risk factors in multivariable models. Since significant interactions were observed between sex and some risk factors (minimum p-value < 0.001), all analyses were done separately in men and women. We did not consider multiple testing to correct levels of significance because our primary objective was to explore potential associations between each variable and incident hypertension [20]. Nevertheless, considering multiple testing in examining the interaction of sex with different potential risk factors in multivariable analysis, the level of p-value was decreased to 0.002 (0.05/24) and significant interactions were still observed between sex with age and marital status.

To reduce selection bias, propensity scores (PS), the estimated probability of being respondents versus non-respondents, were computed using maximum likelihood logistic regression analysis in both sexes and in the whole population. All baseline measures were included in a logistic model as exposures, and participation in the follow-up as the outcome, with the inverse of the PS being used as the sampling weight in the analysis [21]. All analyses were conducted using STATA version 12 SE (StataCorp, TX, USA) and a two-tailed p-value < 0.05 was considered significant.

## Results

The study population consisted of 2894 men and 3639 women at baseline with a mean (SD) age of 40.3 (13.4) and 37.7 (11.5) years, respectively. The baseline characteristics of respondents and non-respondents are shown in Table 1. There were significant differences between the study population (respondents) and non-respondents in both men and women; respondents had higher levels of BMI and TC, and higher frequencies of family history of premature CVD (among men) and low physical activity (among men), whereas non-respondents had higher levels of eGFR (among women) and higher frequencies of T2DM, being a current smoker, using glucose-lowering medications, using lipid-lowering medications, and a positive family history of T2DM.

**Table 1:** Baseline characteristics of the respondents (study participants) and non-respondents: Tehran Lipid and Glucose Study 1999–2018.

		Women		p-value	Men		p-value	p-value*
	
	Total respondents (n = 6533)	Non-Respondents (n = 1720)	Respondents (n = 3639)		Non-Respondents (n = 1377)	Respondents (n = 2894)		

**Age (year)**	38.8(12.5)	37.6(14.2)	37.7(11.5)	0.79	40.0(15.0)	40.3(13.4)	0.42	<0.001
**BMI (kg/m^2^)**	26.3(4.4)	26.5(5.0)	26.9(4.7)	0.005	25.1(4.2)	25.4(3.9)	0.01	<0.001
**WC (cm)**	86.5(11.6)	84.8(12.7)	85.4(12.1)	0.13	87.2(11.7)	87.8(10.8)	0.12	<0.001
**SBP (mmHg)**	112.2(11.4)	110.8(11.5)	110.8(11.5)	0.49	113.5(11.3)	114.0(11.0)	0.26	<0.001
**DBP (mmHg)**	74.0(8.2)	73.5(8.2)	73.8(8.1)	0.25	74.0(8.2)	74.2(8.2)	0.55	0.08
**FPG (mmol/L)**	5.0(0.89)	5.6(2.4)	5.0(0.84)	<0.001	5.7(2.3)	5.1(0.94)	<0.001	<0.001
**2h-PCPG (mmol/L)**	6.0(2.5)	6.1(2.0)	6.2(2.3)	0.13	5.9(2.7)	5.9(2.7)	0.78	<0.001
**eGFR (ml/min/1.73 m^2^)**	78.2(13.6)	78.8(15.2)	77.0(13.2)	<0.001	80.6(17.8)	79.7(13.8)	0.08	<0.001
**TC(mmol/L)**	5.2(1.1)	5.1(1.2)	5.2(1.14)	0.006	5.0(1.07)	5.1(1.11)	<0.001	0.06
**TG (mmol/L)**	1.47(1.18)	1.33(1.06)	1.34(1.04)	0.6	1.58(1.24)	1.66(1.33)	0.04	<0.001
**HDL-C(mmol/L)**	1.08(0.28)	1.15(0.29)	1.16(0.29)	0.13	1.0(0.26)	0.98(0.24)	0.85	<0.001
**Marital status, n (%)**				<0.001			<0.001	<0.001
**– Single**	982(15.0)	349(20.3)	423(11.6)		349(25.3)	559(19.3)		
**– Married**	5311(81.3)	1224(71.2)	2998(82.4)		1013(73.6)	2313(80.0)		
**– Widowed/divorced**	240(3.7)	147(8.5)	219(6.0)		15(1.1)	21(0.7)		
**Smoking status, n (%)**				<0.001			<0.001	<0.001
**– Never**	4999(76.5)	1447(90.7)	3433(94.4)		621(50.5)	1566(54.1)		
**– Former**	422(6.5)	39(2.4)	47(1.3)		115(9.4)	375(13.0)		
**– Current**	1112(17.0)	109(6.8)	159(4.4)		493(40.1)	953(32.9)		
**Education, n (%)**				0.1			0.17	<0.001
**– <6 years**	1650(25.3)	531(31.0)	1039(28.6)		298(21.7)	611(21.1)		
**– 6–12 years**	3860(59.1)	962(56.2)	2156(59.2)		833(60.7)	1704(58.9)		
**– >12 years**	1023(15.7)	220(12.8)	441(12.2)		241(17.6)	579(20.0)		
**Low physical activity, n(%)**	4612(70.6)	932(70.3)	2529(69.5)	0.57	729(68.7)	2083(72.0)	0.045	0.03
**Glycemic status categories, n(%)**				<0.001			<0.001	0.54
**– Normal**	5191(79.5)	965(70.2)	2905(79.8)		658(65.9)	2286(79.0)		
**– Pre-Diabetes**	1081(16.5)	216(15.7)	586(16.1)		160(16.0)	495(17.1)		
**– Diabetes**	261(4.0)	193(14.0)	148(4.1)		181(18.1)	113(3.9)		
**FHDM, n (%)**	1736(26.6)	487(30.3)	1000(27.5)	0.04	353(28.4)	736(25.4)	0.05	0.065
**Family history of premature CVD, n (%)**	968(14.8)	259(15.1)	558(15.3)	0.8	155(11.3)	410(14.2)	0.01	0.19
**Glucose lowering medications, n (%)**	9(0.1)	120(7.0)	3(0.1)	<0.001	91(6.6)	6(0.2)	<0.001	0.18
**Lipid lowering medications, n (%)**	87(1.3)	60(3.5)	63(1.7)	<0.001	25(1.8)	24(0.8)	0.005	0.002
**Hypertension incidence, n (%)**	2131(32.6)	–	1136(31.2)	–	–	995(34.4)	–	0.007

BMI: body mass index; WC: waist circumference; SBP: systolic blood pressure; DBP: diastolic blood pressure; FPG: fasting plasma glucose; 2h-PCPG: 2-hour post-challenge plasma glucose; eGFR: estimated glomerular filtration rate; TC: Total cholesterol; TG: Triglyceride; HDL-C: high-density lipoprotein cholesterol; FHDM: family history of diabetes; CVD: cardiovascular disease. Values are shown as Mean (SD) and number (%), for continuous and categorical variables, respectively; for TG values are shown as Median (Interquartile range). * The comparison p-value between men and women respondents.

During the median (IQR) follow-up of 13.1 years (8.2–16.0), 995 men and 1136 women experienced hypertension. The crude and age-standardized incidence rates (95% CI) of hypertension in the whole population were 27.5 (26.3–28.7) and 36.1 (34.3–38.0) per 1000 person-years; the corresponding sex-specific rates were 29.7 (27.8–31.6) and 34.9 (32.5–37.4) per 1000 person-years in men and 25.8 (24.3–27.3) and 38.7 (35.5–42.0) per 1000 person-years in women, respectively. The highest rate of incident hypertension was in women aged ≥60 years (80.2 per 1000 person-year). Older adults experienced a higher incidence of hypertension but the slope of this increase was steeper from the ’20s to ’30s, about 1.5-fold in men and >2-fold in women (Table 2). The ASRs were also calculated using the most recent Iranian census (2016), with no change in findings (data not shown).

**Table 2:** The crude and age-standardized incidence rate (ASR) (per 1000 person-years) of hypertension by age and sex: Tehran Lipid and Glucose Study (1999–2018).

Age categories, years	Total population			Men			Women		

	E/N	Crude	ASR	E/N	Crude	ASR	E/N	Crude	ASR

**– 20–29**	279/1821	11.7(10.4–13.1)	11.7(10.3–13.1)	146/725	16.1(13.7–18.9)	16.1(13.6–18.9)	133/1096	8.9(7.6–10.6)	8.9(7.5–10.6)
**– 30–39**	563/2083	21.2(19.5–23.0)	21.2(19.5–23.0)	269/913	23.7(21.1–26.8)	23.7(21.0–26.8)	294/1170	19.3(17.2–21.6)	19.3(17.1–21.6)
**– 40–49**	578/1372	37.4(34.4–40.5)	37.4(34.4–40.5)	222/581	33.0(28.9–37.6)	33.0(28.8–37.6)	356/791	40.7(36.9–45.2)	40.7(36.9–45.1)
**– 50–59**	432/775	57.0(51.9–62.6)	57.0(51.7–62.6)	186/361	49.9(43.2–57.6)	49.9(43.0–57.6)	246/414	63.8(56.3–72.3)	63.8(56.1–72.3)
**– ≥60**	279/482	69.6(61.9–78.2)	69.6(61.6–78.2)	172/314	64.3(55.4–74.6)	64.3(55.4–74.6)	107/168	80.2(66.3–96.9)	80.2(65.7–96.9)
**Total**	2131/6533	27.5(26.3–28.7)	36.1(34.3–38.0)	995/2894	29.7(27.8–31.6)	34.9(32.5–37.4)	1136/3639	25.8(24.3–27.3)	38.7(35.5–42.0)

E/N: Event/Number; ASR: Age-standardized rates using Standard world (Segi’s) population.

Sex-specific univariable HRs (95% CI) of continuous and categorical risk factors are shown in Supplementary Tables 1 and 2, respectively.

Sex-specific multivariable-adjusted HRs (95% CI) of continuous risk factors for incident hypertension are presented in Supplementary Table 3. In both men and women, age and baseline SBP/DBP significantly increased the risk of incident hypertension. Moreover, among women, BMI, former smoking and family history of premature CVD increased the risk for hypertension. However, an increase in HDL-C was associated with a lower risk of hypertension among women. The corresponding discrimination power of the multivariable prediction models (Supplementary Table 3) as represented by optimism-corrected corrected Harrell’s C index (95% CI), was 0.76 (0.74–0.77) among men and 0.76 (0.76–0.79) among women.

**Table 3:** Hazard ratios (HR) and 95% confidence intervals (CI) from the multivariable* analysis of categorical potential risk factors in relation to hypertension incidence by sex: Tehran Lipid and Glucose Study (1999–2018).

	Men		Women		Total population		Sex interaction

	HR(95% CI)	p-value	HR(95% CI)	p-value	HR(95% CI)	p-value	p-value

**Sex, women (reference)**			–	–	1.02(0.91–1.14)	0.73	–
**Age categories, years**							**0.002**
**– 20–29**	Reference		Reference		Reference		
**– 30–39**	**1.56(1.20–2.03)**	**0.001**	**1.70(1.36–2.13)**	**<0.001**	**1.61(1.37–1.90)**	**<0.001**	
**– 40–49**	**2.19(1.63–2.95)**	**<0.001**	**2.85(2.24–3.62)**	**<0.001**	**2.49(2.08–2.98)**	**<0.001**	
**– 50–59**	**3.03(2.20–4.16)**	**<0.001**	**3.97(2.98–5.28)**	**<0.001**	**3.38(2.75–4.16)**	**<0.001**	
**– ≥60**	**4.11(2.92–5.80)**	**<0.001**	**4.55(3.15–6.58)**	**<0.001**	**4.16(3.26–5.29)**	**<0.001**	
**BMI categories, kg/m**^2^							**0.053**
**– <25**	Reference		Reference		Reference		
**– 25–30**	1.15(0.97–1.36)	0.12	**1.20(1.00–1.44)**	**0.05**	**1.19(1.05–1.34)**	**0.006**	
**– ≥30**	**1.39(1.05–1.83)**	**0.02**	**1.58(1.25–1.99)**	**<0.001**	**1.53(1.28–1.82)**	**<0.001**	
**Central obesity, yes**	**1.26(1.03–1.54)**	**0.025**	1.11(0.91–1.35)	0.3	**1.17(1.02–1.35)**	**0.025**	0.41
**Blood pressure categories**							0.13
**– Optimum**	Reference		Reference		Reference		
**– Normal**	**2.32(1.97–2.74)**	**<0.001**	**2.54(2.18–2.96)**	**<0.001**	**2.43(2.17–2.72)**	**<0.001**	
**– High normal**	**4.17(3.49–4.97)**	**<0.001**	**4.65(3.91–5.34)**	**<0.001**	**4.43(3.91–5.03)**	**<0.001**	
**Marital status**							0.001
**– Single**	Reference		Reference		Reference		
**– Married**	0.83(0.63–1.08)	0.17	1.30(0.95–1.77)	0.1	0.92(0.77–1.11)	0.38	
**– Widowed/divorced**	0.75(0.33–1.71)	0.49	1.39(0.94–2.05)	0.1	0.99(0.74–1.33)	0.96	
**Smoking status**							0.26
**– Never**	Reference		Reference		Reference		
**– Former**	1.03(0.85–1.25)	0.73	**2.15(1.52–3.04)**	**<0.001**	1.12(0.93–1.34)	0.23	
**– Current**	0.98(0.84–1.15)	0.82	0.99(0.73–1.33)	0.94	0.93(0.88–1.16)	0.89	
**Education level, years**							0.13
**– <6**	Reference		Reference		Reference		
**– 6–12**	1.04(0.93–1.24)	0.62	0.98(0.84–1.15)	0.84	0.98(0.87–1.11)	0.8	
**– >12**	0.91(0.72–1.14)	0.42	0.97(0.75–1.27)	0.85	0.88(0.74–1.04)	0.13	
**Low physical activity, yes**	–	–	1.06(0.90–1.24)	0.48	–	–	
**Hypercholesterolemia, yes**	1.09(0.94–1.26)	0.27	0.89(0.77–1.04)	0.14	1.0(0.91–1.12)	0.89	0.8
**Low HDL-C, yes**	1.07(0.92–1.25)	0.39	1.06(0.90–1.24)	0.48	1.05(0.95–1.18)	0.33	0.9
**Hypertriglyceridemia, yes**	1.00(0.85–1.16)	0.93	**1.23(1.06–1.43)**	**0.005**	**1.11(1.00–1.24)**	**0.05**	**0.003**
**Glycemic status categories**							**0.01**
**– Normal**	Reference		Reference		Reference		
**– Pre-diabetes**	0.93(0.78–1.10)	0.40	**1.19(1.02–1.39)**	0.03	1.03(0.91–1.16)	0.62	
**– Diabetes**	0.91(0.66–1.25)	0.56	1.09(0.79–1.50)	0.61	0.98(0.78–1.23)	0.85	
**CKD, yes**	0.97(0.75–1.25)	0.8	1.07(0.86–1.32)	0.55	1.04(0.89–1.23)	0.60	0.24
**Family history of premature CVD, yes**	1.12(0.92–1.37)	0.24	1.15(0.95–1.38)	0.14	1.14(0.99–1.30)	0.06	0.78

* Weighted by inverse probability of propensity score to adjust for baseline differences between responders and non-responders. BMI: body mass index; HDL-C: high-density lipoprotein cholesterol; CKD: chronic kidney disease; CVD: cardiovascular disease.

Table 3 shows the multivariable Cox regression of all significant categorical risk factors. In line with continuous analysis, among men and women, age groups (all defined decades), hypertension categories including normal and high normal blood pressure (optimum as reference) increased the risk of incident hypertension. Moreover, among women, a BMI greater than 25 kg/m^2^, former smoking, hypertriglyceridemia and pre-DM and among men, central obesity and BMI ≥30 kg/m^2^ increased the risk of incident hypertension. The optimism-corrected Harrell’s C index (95% CI) in the categorical adjusted model was 0.75 (0.74–0.79) among men and 0.75 (0.74–0.76) among women.

## Discussion

In our study – a cohort with a median follow-up of 13 years – nearly 2.7% of total participants (3% of men and 2.6% of women) developed hypertension, annually. Among those who were less than 40 years old, the incidence rate of hypertension was more prominent in men; however, among older age groups, the incidence of hypertension was more pronounced in women. Moreover, the incidence rate increased with aging in both sexes, with a steeper increase slope among younger age groups. In addition to aging, being generally obese and having a baseline BP≥120/80 mmHg were significant risk factors in both sexes. Being overweight, being a former smoker, having prediabetes status, and high TG in women and central obesity in men were also independent risk factors. Finally, a family history of premature CVD was associated with a 14% increased risk of hypertension in the total population, which was marginally significant.

The crude incidence rate of hypertension was in line with findings from the short-term follow-up of the TLGS [3] in which contrary to the current study, members of the population with diabetes at baseline had been excluded. In a study from Isfahan (central zone of Iran), among the population aged ≥35 years, the incidence rate of hypertension was 31.6 per 1000 person-years [8]. Based on the Arici, et al. [9] study from Turkey, another country in the MENA region, the incidence rate of hypertension was 53.6 per 1000 person-years. In two large population-based studies conducted in US and British populations aged ≥35 years, the incidence rates during 9- and 5-year follow-ups were 36.6 and 27.7 per 1000 person-years, respectively [22,23]; rates which were lower than similar age groups in the present study. In a retrospective Korean cohort, the incidence rates of hypertension were 17.4 and 13.9 per 1000 person-years in men and women, respectively, which is apporoximately 50% lower than our findings [24].

The findings of this study showed that a one year increase in age leads to a 3% increase in the risk of hypertension. Aging is a well-known independent risk factor for hypertension [8,9,22,24,25], mostly due to vascular stiffness [26]. Likewise, we found that the incidence rate in younger age groups was lower among women, whereas after the fourth decade, this rate was higher in women. Concordant with this finding, the Framingham Heart Study [27] and some other cohort studies in different countries [24,28,29] reported this sex-age crossover. Among the different risk factors, the effect modification of sex was significant for age at the level of p-value < 0.001. This crossover pattern might be related to loss of the protective effect of estrogen after menopause, which in turn leads to hypertension [30]. Moreover, it was shown that hypertensive postmenopausal women were more salt-sensitive compared to controls; this issue being attributable in part to the loss of the modulating impact of sex steroids on tubular sodium reabsorption [30]. Despite this, in agreement with a previous study [25], we did not find sex to be an independent risk factor for incident hypertension. Some previous studies have reported the male sex [8,31] and others, the female sex [22,32] to be a risk factor for hypertension.

Obesity has been identified as a major risk factor for hypertension development through induced insulin resistance (IR), low-grade inflammatory status, and activation of the sympathetic nervous system [33]. In keeping with other cohort studies and a meta-analysis [8,28,33], the results of this study showed that both indices of general and central obesity were independently associated with incident hypertension. In our data analysis, we did not find a significant interaction between general obesity and sex at the level of p = 0.002; however, being overweight and obese was associated with a greater risk of hypertension among women rather than men, as shown in Table 3. It was shown that a comparable increase in BMI causes a greater increase in SBP among women than men. However, why obesity has a more adverse effect on BP among women has not yet been explored [30].

IR has a major role in the pathogenesis of hypertension [34]. Previously, we found that women with the highest level of IR have an 80% higher risk of incident hypertension [34]. In the current study, prediabetes state, as a surrogate for IR, was significantly associated with hypertension development among women (p-value for the interaction of all glucose tolerance categories with sex = 0.01). Moreover, consistent with our previous short-term follow-up [3], hypertriglyceridemia (in categorical analysis) and low HDL-C (in continuous analysis) remained independent risk factors, but only among women. A possible explanation for this might be related to the association of the TG/HDL-C ratio with incident IR in the study population [35].

A previous systematic review found that baseline SBP and DBP is the most constant and strongest component of hypertension predicting models, regardless of whether it is considered a continuous or a categorized variable [4]. In a national survey, it was shown that 39.8% of Iranian subjects aged 25–70 years were prehypertensive [2]. Bozorgmanesh et al. [36] reported that 5.6% of Iranian prehypertensive individuals develop hypertension annually. These findings and our results for SBP and DBP warn that preventive programs should focus on individuals with prehypertension.

We found a significant and strong interaction between sex and marital status. Although marital status was not a significant risk factor for hypertension development in this study, in line with a recent study [37], we found that married men had a 17% lower and married women had a 30% higher risk of incident hypertension. It is suggested that married men have better sleep patterns and lifestyle compared to never-married men [37].

Despite the logical concept that smoking leads to arterial stiffness and ultimately hypertension, the association betweem current smoking with incident hypertension is controversial [38]. In the current study, we did not detect any evidence for the impact of current smoking on incident hypertension; however, being a former smoker was shown to be a risk factor for hypertension among women. This issue might be justified by weight gain after smoking cessation [39]. Meta-analyses have shown that the impact of smoking on incident CHD and stroke is higher among women than men; however, this study has been unable to demonstrate any significant interaction between smoking and sex [30].

The strengths of this study consist of having a large population-based sample with a long duration of follow-up, standardized measurement techniques and assessment of a wide range of possible risk factors. A number of important limitations need to be considered. First, the sample was limited to a metropolitan city and cannot be generalized to a rural population. Second, we relied on office BP measurements rather than ambulatory BP measurements; hence, we cannot diagnose those with ‘white coat’ hypertension or ‘masked’ hypertension [40]; however, we tried to minimize this limitation by doing two standard measurements. Third, risk factors were only considered at the time of baseline recruitment and possible changes in risk factor status during the follow-up period were not taken into account. Finally, we did not have access to some potential predictors of hypertension such as genetic susceptibility, air pollution, nutritional status and family history of hypertension [4,6,41] in the present study.

## Conclusion

We found that in a Middle Eastern adult population, during over 13-year follow-up, the age-standardized incidence rate of hypertension was 36.1 per 1000 person-years. Moreover, among non-modifiable risk factors, age and family history of premature CVD (among women) were risk factors for incident hypertension. Also regarding modifiable risk factors, obesity status, high BP levels in both sexes, and hypertriglyceridemia, prediabetes status and former smoking in women, were important risk factors for incident hypertension. Our data analysis has certain clinical implications, namely that for the primary prevention of hypertension, more attention should be paid to sex-specific evaluation of potential risk factors. This is particularly true of hypertriglyceridemia and prediabetes, which are both important surrogates for insulin resistance.

## Additional Files

The additional files for this article can be found as follows:

10.5334/gh.780.s1 Supplementary Table 1. Hazard ratios (HR) and 95% confidence intervals (CI) from the univariable analysis of continuous potential risk factors in relation to hypertension incidence by sex: Tehran Lipid and Glucose Study (1999–2018).

10.5334/gh.780.s2 Supplementary Table 2. Hazard ratios (HR) and 95% confidence intervals (CI) from the univariable analysis of categorical potential risk factors in relation to hypertension incidence by sex: Tehran Lipid and Glucose Study (1999–2018).

10.5334/gh.780.s3 Supplementary Table 3. Hazard ratios (HR) and 95% confidence intervals (CI) from the multivariable analysis* of continuous potential risk factors in relation to hypertension incidence by sex: Tehran Lipid and Glucose Study (1999–2018).

## Abbreviations

Type 2 diabetes mellitus (T2DM)

Middle East and North Africa (MENA)

Tehran Lipid and Glucose Study (TLGS)

Body mass index (BMI)

Waist circumference (WC)

Fasting plasma glucose (FPG)

2-hour post-challenge plasma glucose (2h-PCPG)

High density lipoprotein cholesterol (HDL-C)

Systolic blood pressure (SBP)

Diastolic blood pressure (DBP)
