# Correction To: Sex-Specific Incidence Rates and Risk Factors for Hypertension During 13 Years of Follow-up: The Tehran Lipid and Glucose Study

**DOI:** 10.5334/gh.847

**Published:** 2020-07-29

**Authors:** Samaneh Asgari, Seyyed Saeed Moazzeni, Fereidoun Azizi, Hengameh Abdi, Davood Khalili, Monir Sadat Hakemi, Farzad Hadaegh

**Affiliations:** 1 Prevention of Metabolic Disorders Research Center, Research Institute for Endocrine Sciences, Shahid Beheshti University of Medical Sciences, Tehran, IR; 2 Nephrology Ward of Shariati Hospital, Tehran University of Medical Sciences, Tehran, IR; 3 Endocrine Research Center, Research Institute for Endocrine Sciences, Shahid Beheshti University of Medical Sciences, Tehran, IR; 4 Department of Epidemiology and Biostatistics, Research Institute for Endocrine Sciences, Shahid Beheshti University of Medical Sciences, Tehran, IR

## Abstract

Corrigendum In the original version of the article, the affiliation of Samaneh Asgari, Seyyed Saeed Moazzeni, Davood Khalili, and Farzad Hadaegh is not complete. The affiliation needs to be corrected to: Prevention of Metabolic Disorders Research Center, Research Institute for Endocrine Sciences, Shahid Beheshti University of Medical Sciences, Tehran, Iran. Published article information: Asgari S, Moazzeni SS, Azizi F, Abdi H, Khalili D, Hakemi MS, Hadaegh F. Sex-Specific Incidence Rates and Risk Factors for Hypertension During 13 Years of Follow-up: The Tehran Lipid and Glucose Study. *Global Heart*. 2020; 15(1). doi: 10.5334/gh.780 PMCID: PMC7218790 PMID: 32489802.

Editor-in-chief

Global Heart

Dear prof. Diederick E. Grobbee,

Greetings,

In the original version of the article, the affiliation of Samaneh Asgari, Seyyed Saeed Moazzeni, Davood Khalili, and Farzad Hadaegh is not complete.

The affiliation needs to be corrected to:

Prevention of Metabolic Disorders Research Center, Research Institute for Endocrine Sciences, Shahid Beheshti University of Medical Sciences, Tehran, Iran.

**Published article information**

Asgari S, Moazzeni SS, Azizi F, Abdi H, Khalili D, Hakemi MS, Hadaegh F. Sex-Specific Incidence Rates and Risk Factors for Hypertension During 13 Years of Follow-up: The Tehran Lipid and Glucose Study. *Global Heart*. 2020; 15(1).

doi: 10.5334/gh.780

PMCID: PMC7218790

PMID: 32489802

Yours sincerely

Farzad Hadaegh, MD

