# Supplementary material for: Sex-Specific Incidence Rates and Risk Factors for Hypertension During 13 Years of Follow-up: The Tehran Lipid and Glucose Study
Source: Glob Heart. 2020 Apr 8;15(1):29. doi: 10.5334/gh.780 (PMC7218790; doi:10.5334/gh.780)
Supplement: Supplementary Table 1. — Hazard ratios (HR) and 95% confidence intervals (CI) from the univariable analysis of continuous potential risk factors in relation to hypertension incidence by sex: Tehran Lipid and Glucose Study (1999–2018). [file gh-15-1-780-s1.pdf]

†Supplementary Table 1: Hazard ratios (HR) and 95% confidence intervals (CI) from the univariable analysis of continuous potential risk factors in relation to hypertension incidence by sex: Tehran Lipid and Glucose Study (1999–2018)

	Men		Women		Total population	
	**HR(95% CI)**	**p-value**	**HR(95% CI)**	**p-value**	**HR(95% CI)**	**p-value**
**Age (year)**	1.04(1.03-1.04)	<0.001	1.06(1.06-1.07)	<0.001	1.05(1.04-1.05)	<0.001
**BMI (kg/m^2)**	1.09(1.08-1.11)	<0.001	1.11(1.10-1.13)	<0.001	1.10(1.09-1.11)	<0.001
**WC (cm)**	1.04(1.03-1.04)	<0.001	1.05(1.04-1.06)	<0.001	1.05(1.04-1.05)	<0.001
**SBP (mmHg)**	1.07(1.06-1.07)	<0.001	1.08(1.08-1.09)	<0.001	1.07(1.07-1.08)	<0.001
**DBP (mmHg)**	1.07(1.06-1.08)	<0.001	1.10(1.09-1.11)	<0.001	1.09(1.08-1.09)	<0.001
**FPG (mmol/L)**	1.13(1.08-1.19)	<0.001	1.27(1.23-1.32)	<0.001	1.20(1.17-1.24)	<0.001
**2h-PCPG(mmol/L)**	1.07(1.05-1.08)	<0.001	1.14(1.12-1.16)	<0.001	1.10(1.08-1.11)	<0.001
**eGFR (ml/min/1.73 m^2)**	0.97(0.97-0.98)	<0.001	0.96(0.96-0.97)	<0.001	0.97(0.96-0.97)	<0.001
**TC (mmol/L)**	1.20(1.14-1.26)	<0.001	1.36(1.30-1.41)	<0.001	1.29(1.25-1.33)	<0.001
**TG (mmol/L)**	1.12(1.08-1.15)	<0.001	1.35(1.30-1.41)	<0.001	1.18(1.16-1.21)	<0.001
**HDL-C(mmol/L)**	0.91(0.70-1.18)	<0.001	0.62(0.50-0.77)	<0.001	0.68(0.58-0.80)	<0.001

BMI: body mass index; WC: waist circumference; SBP: systolic blood pressure; DBP: diastolic blood pressure; FPG: fasting plasma glucose; 2h-PCPG: 2-hour post-challenge plasma glucose; eGFR: estimated glomerular filtration rate; TC: Total cholesterol; TG: Triglyceride; HDL-C: high-density lipoprotein cholesterol.
