# Supplementary material for: Sex-Specific Incidence Rates and Risk Factors for Hypertension During 13 Years of Follow-up: The Tehran Lipid and Glucose Study
Source: Glob Heart. 2020 Apr 8;15(1):29. doi: 10.5334/gh.780 (PMC7218790; doi:10.5334/gh.780)
Supplement: Supplementary Table 2. — Hazard ratios (HR) and 95% confidence intervals (CI) from the univariable analysis of categorical potential risk factors in relation to hypertension incidence by sex: Tehran Lipid and Glucose Study (1999–2018). [file gh-15-1-780-s2.pdf]

Supplementary Table 2: Hazard ratios (HR) and 95% confidence intervals (CI) from the univariable analysis of categorical potential risk factors in relation to hypertension incidence by sex: Tehran Lipid and Glucose Study (1999–2018)

	Men		Women		Total population	
	**HR(95% CI)**	**p-value**	**HR(95% CI)**	**p-value**	**HR(95% CI)**	**p-value**
**Sex, women (reference)**	-	-	-	-	1.15(1.06-1.26)	0.001
**Age categories, years**						
- **20-29**	Reference		Reference		Reference	
- **30-39**	1.48(1.21-1.81)	<0.001	2.16(1.76-2.65)	<0.001	1.82(1.58-2.10)	<0.001
- **40-49**	2.07(1.68-2.55)	<0.001	4.71(3.89-5.76)	<0.001	3.27(2.83-2.77)	<0.001
- **50-59**	3.18(2.56-3.95)	<0.001	7.59(6.14-9.38)	<0.001	5.10(4.38-5.92)	<0.001
- **≥60**	4.20(3.37-5.24)	<0.001	9.93(7.70-12.83)	<0.001	6.40(5.42-7.56)	<0.001
**BMI categories, kg/m^2**						
- **<25**	Reference		Reference		Reference	
- **25-30**	1.56(1.36-1.79)	<0.001	2.14(1.82-2.51)	<0.001	1.76(1.59-1.96)	<0.001
- **≥30**	2.35 (1.96-2.81)	<0.001	3.82(3.25-4.49)	<0.001	2.88(2.57-3.23)	<0.001
**Central obesity, yes**	1.92(1.68-2.19)	<0.001	2.80(2.48-3.16)	<0.001	2.35(2.15-2.58)	<0.001
**Blood pressure categories**						
- **Optimum**	Reference		Reference		Reference	
- **Normal**	2.43(2.09-2.83)	<0.001	3.33(2.90-3.82)	<0.001	2.90(2.62-3.21)	<0.001
- **High normal**	4.67(4.0-5.45)	<0.001	7.01(6.04-8.13)	<0.001	5.81(5.22-6.46)	<0.001
**Hypercholesterolemia, yes**	1.48(1.30-1.67)	<0.001	1.98(1.76-2.24)	<0.001	1.73(1.59-1.89)	<0.001
**Low HDL-C, yes**	1.05(0.92-1.20)	0.45	1.19(1.04-1.37)	0.01	1.10(1.00-1.21)	0.04
**Hypertriglyceridemia, yes**	1.36(1.19-1.54)	<0.001	2.35(2.09-2.64)	<0.001	1.84(1.69-2.00)	<0.001
**Marital status**						
- **Single**	Reference		Reference		Reference	
- **Married**	1.79(1.49-2.16)	<0.001	3.09(2.33-4.10)	<0.001	2.11(1.81-2.46)	<0.001

- **Widowed/ divorced**	1.90(0.89-4.07)	0.1	5.71(4.07-8.02)	<0.001	3.47(2.73-4.41)	<0.001
**Smoking status**						
- **Never**	Reference		Reference		Reference	
- **Former**	1.18(0.99-1.42)	0.07	2.72(1.86-4.00)	<0.001	1.45(1.24-1.70)	<0.001
- **Current**	0.83(0.72-0.96)	0.01	0.91(0.67-1.24)	0.55	0.93(0.82-1.07)	0.23
**Education, years**						
- **<6**	Reference		Reference		Reference	
- **6-12**	0.6(0.52-0.70)	<0.001	0.39(0.35-0.44)	<0.001	0.47(0.43-0.52)	<0.001
- **>12**	0.56(0.46-0.68)	<0.001	0.24(0.21-0.38)	<0.001	0.43(0.38-0.50)	<0.001
**Low physical activity ,yes**	0.99(0.86-1.15)	0.95	1.14(1.0-1.30)	0.05	1.08(0.98-1.19)	0.36
**Glycemic status categories**						
- **Normal**	Reference		Reference		Reference	
- **Pre-diabetes**	1.52(1.31-1.77)	<0.001	2.05(1.78-2.36)	<0.001	1.79(1.61-1.98)	<0.001
- **Diabetes**	1.63(1.22-2.19)	0.001	2.24(2.60-4.04)	<0.001	2.42(2.03-2.88)	<0.001
**CKD, yes**	2.14(1.75-2.62)	<0.001	2.58(2.21-3.01)	<0.001	2.36(2.01-2.67)	<0.001
**FHDM, yes**	1.01(0.88-1.17)	0.86	1.07(0.94-1.22)	0.27	1.04(0.95-1.15)	0.27
**Family history of premature CVD, yes**	1.13(0.96-1.35)	0.13	1.36(1.17-1.58)	<0.001	1.25(1.12-1.40)	<0.001

BMI: body mass index; HDL-C: high-density lipoprotein cholesterol; CKD: chronic kidney disease; CVD: cardiovascular disease; FHDM: family history of diabetes.
