# Supplementary material for: Sex-Specific Incidence Rates and Risk Factors for Hypertension During 13 Years of Follow-up: The Tehran Lipid and Glucose Study
Source: Glob Heart. 2020 Apr 8;15(1):29. doi: 10.5334/gh.780 (PMC7218790; doi:10.5334/gh.780)
Supplement: Supplementary Table 3. — Hazard ratios (HR) and 95% confidence intervals (CI) from the multivariable analysis* of continuous potential risk factors in relation to hypertension incidence by sex: Tehran Lipid and Glucose Study (1999–2018). [file gh-15-1-780-s3.pdf]

Supplementary Table 3: Hazard ratios (HR) and 95% confidence intervals (CI) from the multivariable analysis* of continuous potential risk factors in relation to hypertension incidence by sex: Tehran Lipid and Glucose Study (1999–2018)

	Men		Women		Total population		Sex interaction
	**HR(95% CI)**	**p-value**	**HR(95% CI)**	**p-value**	**HR(95% CI)**	**p-value**	**p-value**
**Sex, women (reference)**	-	-	-	-	0.92(0.81-1.05)	0.22	
**Age, years**	**1.03(1.02-1.04)**	**<0.001**	**1.04(1.03-1.05)**	**<0.001**	**1.03(1.02-1.04)**	**<0.001**	**<0.001**
**BMI (kg/m^2)**	1.03(0.99-1.07)	0.1	**1.03(1.01-1.06)**	**0.004**	**1.03(1.01-1.05)**	**0.002**	0.35
**WC (cm)**	1.00(0.99-1.02)	0.32	1.00(0.99-1.02)	0.18	**1.01(1.00-1.02)**	**0.05**	0.065
**SBP (mmHg)**	**1.05(1.04-1.05)**	**<0.001**	**1.04(1.04-1.05)**	**<0.001**	**1.04(1.04-1.05)**	**<0.001**	0.096
**DBP (mmHg)**	**1.03(1.02-1.04)**	**<0.001**	**1.04(1.03-1.05)**	**<0.001**	**1.04(1.03-1.05)**	**<0.001**	0.23
**FPG (mmol/L)**	0.99(0.88-1.10)	0.81	0.96(0.85-1.08)	0.5	0.97(0.89-1.06)	0.52	0.083
**2h-PCPG (mmol/L)**	1.01(0.97-1.04)	0.78	1.03(0.99-1.08)	0.15	1.01(0.99-1.05)	0.29	0.06
**eGFR (ml/min/1.73 m^2)**	0.99(0.99-1.00)	0.28	1.0(0.99-1.01)	0.59	1.0(0.99-1.00)	0.2	0.12
**TC (mmol/L)**	0.97(0.89-1.05)	0.42	0.94(0.88-1.01)	0.1	0.97(0.92-1.02)	0.27	0.91
**TG (mmol/L)**	1.05(0.99-1.11)	0.12	0.99(0.91-1.06)	0.63	1.02(0.97-1.07)	0.44	0.94
**HDL-C (mmol/L)**	1.11(0.78-1.57)	0.55	**0.67(0.53-0.86)**	**0.002**	0.84(0.69-1.02)	0.08	**0.03**
**Marital status**							**0.003**
- **Single**	Reference		Reference		Reference		
- **Married**	0.87(0.70-1.10)	0.26	1.16(0.86-1.59)	0.33	0.92(0.77-1.09)	0.35	
- **Widowed/ divorced**	0.81(0.40-1.67)	0.58	1.17(0.79-1.73)	0.44	0.97(0.73-1.29)	0.83	

**Smoking status**							0.62
- **Never**	Reference		Reference		Reference		
- **Former**	1.04(0.85-1.26)	0.71	**1.78(1.21-2.61)**	**0.003**	1.11(0.92-1.33)	0.27	
- **Current**	1.05(0.89-1.22)	0.57	1.08(0.81-1.43)	0.6	1.09(0.95-1.25)	0.22	
**Education level**							**0.046**
- **<6 years**	Reference		Reference		Reference		
- **6-12 years**	1.08(0.89-1.29)	0.43	1.02(0.87-1.20)	0.77	1.02(0.91-1.15)	0.73	
- **>12 years**	0.90(0.72-1.13)	0.36	0.99(0.76-1.30)	0.96	0.89(0.75-1.06)	0.18	
**Family history of premature CVD, yes**	1.13(0.93-1.38)	0.22	**1.20(1.02-1.41)**	**0.03**	**1.16(1.02-1.32)**	**0.023**	0.82

*Weighted by inverse probability of propensity score to adjust for baseline differences between responders and non-responders.

BMI: body mass index; WC: waist circumference; SBP: systolic blood pressure; DBP: diastolic blood pressure; FPG: fasting plasma glucose; 2h-PCPG: 2-hour post-challenge plasma glucose; eGFR: estimated glomerular filtration rate; TC: Total cholesterol; TG: Triglyceride; HDL-C: high-density lipoprotein cholesterol; CVD: cardiovascular disease.
